# Microfluidic-Assisted Preparation of 5-Fluorouracil-Loaded PLGA Nanoparticles as a Potential System for Colorectal Cancer Therapy

**DOI:** 10.3390/ma13071483

**Published:** 2020-03-25

**Authors:** Mahtab Ghasemi Toudeshkchouei, Payam Zahedi, Amin Shavandi

**Affiliations:** 1 Department of Polymer, School of Chemical Engineering, College of Engineering, University of Tehran, P.O. Box 11155-4563, Tehran 1417613131, Iran; mahtab_ghasemi@alumni.ut.ac.ir; 2 BioMatter Unit-Biomass Transformation Lab (BTL), École Interfacultaire de Bioingénieurs (EIB), École Polytechnique de Bruxelles, Université Libre de Bruxelles, Avenue F.D. Roosevelt, 50-CP 165/61, 1050 Brussels, Belgium

**Keywords:** microfluidics, fluorouracil, PLGA, Caco2, cell death, nanoparticle

## Abstract

This work aims at fabricating 5-fluorouracil (5-FU)-loaded poly (lactic-co-glycolic) acid nanoparticles (PLGA NPs) using a microfluidic (MF) technique, with potential for use in colorectal cancer therapy. In order to achieve 5-FU-loaded NPs with an average diameter of approximately 119 nm, the parameters of MF process with fork-shaped patterns were adjusted as follows: the ratio of polymer to drug solutions flow rates was equal to 10 and the solution concentrations of PLGA as carrier, 5-FU as anti-cancer drug and poly (vinyl alcohol) (PVA) as surfactant were 0.2 (% *w*/*v*), 0.01 (% *w*/*v*) and 0.15 (% *w*/*v*), respectively. In this way, a drug encapsulation efficiency of approximately 95% into the PLGA NPs was obtained, due to the formation of a hydrodynamic flow focusing phenomenon through the MF chip. A performance evaluation of the NP samples in terms of the drug release, cytotoxicity and cell death was carried out. Finally, by analyzing the results after induction of cell death and 4′, 6-diamidino-2-phenylin-dole (DAPI) staining, MF-fabricated NPs containing 5-FU [0.2 (% *w*/*v*) of PLGA] revealed the dead cell amounts of 10 and 1.5-fold higher than the control sample for Caco2 and SW-480, respectively.

## 1. Introduction

Colorectal cancer (CRC) is the leading cause of cancer-related mortality and results in around 655,000 deaths worldwide every year [1]. It has been found that a wide range of anticancer drugs, such as 5-fluorouracil (5-FU), has an extensive function in the treatment of CRCs, pancreas, liver, etc. Besides severe side effects in patients, the high hydrophilicity and low half-life of these drugs result in a reduction of their efficacy in oral and intravenous treatments [2,3,4]. 5-FU—as an antimetabolite—can suppress cancer cell growth by repairing deoxyribonucleic acid (DNA) [5]. Because of the many obstacles that conventional anticancer drugs like 5-FU are involved, a large number of researchers have endeavored to employ a wide range of biodegradable and biocompatible polymers for controlled release of 5-FU. Poly (ε-caprolactone) (PCL)/chitosan blend [6], poly (lactic-co-glycolic) acid (PLGA) [7,8], poly (butylene adipate)/terephthalate [9], poly (methyl methacrylate) [10], poly (N-isopropylacrylamide-co-acrylic acid) [11] are abundantly used biomaterials in the field. Amid these polymers, PLGA—a well-known copolymer approved by the US food and drug administration (FDA)—has been extensively employed for the development of drug delivery systems based on micro- and nano-sized particles. PLGA drug carriers can enhance the pharmaceutical/therapeutic characteristics of anticancer agents by prolonging their circulation time, targeting cancer tissues, protecting the drug from rapid elimination/premature degradation [12,13].

Nowadays, nanotechnology is opening new windows in the field of drug delivery for the development of diverse types of nanoparticles (NPs) as carriers based on organic and inorganic materials. In this regard, NPs synthesized from biodegradable polymers have attracted considerable attention owing to their remarkable use in pharmaceutics and biomedicine. In this regard, Ashwanikumar et al. [14] investigated the anticancer performance of 5-FU-loaded PLGA-grafted branched polyethyleneimine NPs on colon cancer cell line (HCT 116). They demonstrated that the fabricated NPs had an excellent cellular uptake capacity. In another study, pH-responsive, magnetic PLGA NPs loaded with 5-FU were efficient for the treatment of CT26 colon tumors in specific kinds of mice (BALB/c) [15].

Moreover, Gupta et al. [16] fabricated 5-FU-loaded hybrid nanofibers based on poly (lactic acid) (PLA) and PCL. Their results showed that the effect of polymer matrix type on drug release was an important parameter using biological responses fluorescent imaging and thiazolyl blue (MTT) assay. Although the previous research works articulated that the polymeric samples containing 5-FU with different topographies were efficient on cancerous cells in vitro and in vivo examinations, the utilized processes possessed some limits; for instance, they were time-consuming and relatively cost-effective. Likewise, low precision and reproducibility, as well as a broad polydispersity index (PDI) of particles were other restrictive aspects of mentioned methods.

To overcome these problems, microfluidic (MF) systems with the ability to manipulate and controlling fluids in the range of picoliters to microliters have turned into emerging and efficient tools for the improvement of drug delivery systems. Compared to conventional batch methods, the MF setup has many advantages: better control of material characteristics, precisely controlled release profiles of payloads and high reproducibility, in addition to performing multiple and concurrent assays on the chip [17,18,19,20]. Thus, studies of MF systems as a nanosuspension bottom-up approach have focused increasingly on the fabrication of drug carriers and direct drug delivery to a targeted tissue [21,22,23]. For this reason, the early study on 5-FU release in the MF system with cross-junction microchannel showed that the drug-loaded genipin-gelatin microcapsules produced using this technique had a narrow size distribution with a particle diameter in the range of 130–580 µm [24]. Moreover, Xue et al. [25] worked on the production of 5-FU-loaded biocompatible poly (ethylene glycol) diacrylate microspheres with MF device pattern of T-junction channel that resulted in a monodispersed size distribution from 16.7 to 85.7 µm in diameter, leading to utilizing for sustained drug release. Wang and coworkers [26] synthesized a dual drug-loaded magnetic alginate microsphere containing 5-FU and doxorubicin hydrochloride using MFs, combined with an external ionic crosslinking. Their results exhibited a uniform size of microspheres, excellent vision under magnetic resonance and regular spherical shape. Therefore, the characterized dual drug-loaded microspheres could be used in the integration of chemotherapy diagnosis with a combined modality potentially.

On the other hand, a recent work on dual drug-loaded microspheres based on hypromellose acetate succinate polymer was carried out by Maher et al. [27]. They used MF-based droplets incorporated 5-FU and curcumin into a pH-responsive and magnetic polymeric carrier. In this work, the safety of samples was assessed by using RAW264.7, a murine monocyte/macrophage cell line, the outcomes revealed that the safety of drug-free microspheres was up to 1000 µg/mL. Finally, the researchers showed the synergistic effect of 5-FU and curcumin on SW-480 colon cell line.

Few studies on 5-FU-loaded biocompatible polymer-carriers drawing on MF techniques have been found in literature, especially in terms of evaluating the MF technique for colon cancer cells. This work aims at fabricating 5-FU-loaded PLGA NPs via MF techniques and evaluating their impact on colon cancer cell line death. For this purpose, samples are characterized in terms of size distribution, morphology, drug release, cytotoxicity and cell death.

## 2. Materials and Methods

### 2.1. Materials

Poly (lactic-co-glycolic acid) [PLGA, trade name of Purasorb^®^PLG8523, lactide: glycolide monomer molar ratio of 50:50] was purchased from Purac Co. (Gorinchem, The Netherlands). 5-Fluorouracil [5-FU, C**_4_**H**_3_**FN**_2_**O**_2_**, with a molecular weight of 130.08 g/mol, an anti-cancer drug, white, odorless and crystalline powder] and poly (vinyl alcohol) [PVA, weight average molecular weight of 60,000 g/mol, the hydrolysis degree >98% and a viscosity of 11 mPa-s for the solution (4%) at 20 °C] were purchased from Merck Co. (Darmstadt, Germany). Two colon cancer cell lines, namely Caco2 (extracted from a metastatic tumor) and SW-480 (extracted from a primary tumor), were obtained from the Stem Cell Technology Research Center (Bonyakhteh), Tehran, Iran. Dimethyl sulfoxide (DMSO) was obtained from Sigma-Aldrich (Zwijndrecht, The Netherlands). Knockout Dulbecco’s modified Eagle’s medium (DMEM) with high glucose, Roswell Park Memorial Institute medium (RPMI-1640); trypsin, phosphate-buffered saline (PBS) and fetal bovine serum (FBS) were purchased from Gibco Bio-Cult (Paisley, UK). 3-[4–dimethylthiazol-2-yl]-diphenyl tetrazolium bromide (MTT) and antibody markers were obtained from Sigma-Aldrich (Hamburg, Germany). The other chemicals were analytical reagent grades (Merck Co., Darmstadt, Germany) and were used without further purification.

### 2.2. MF Chip Design

**Figure 1:**
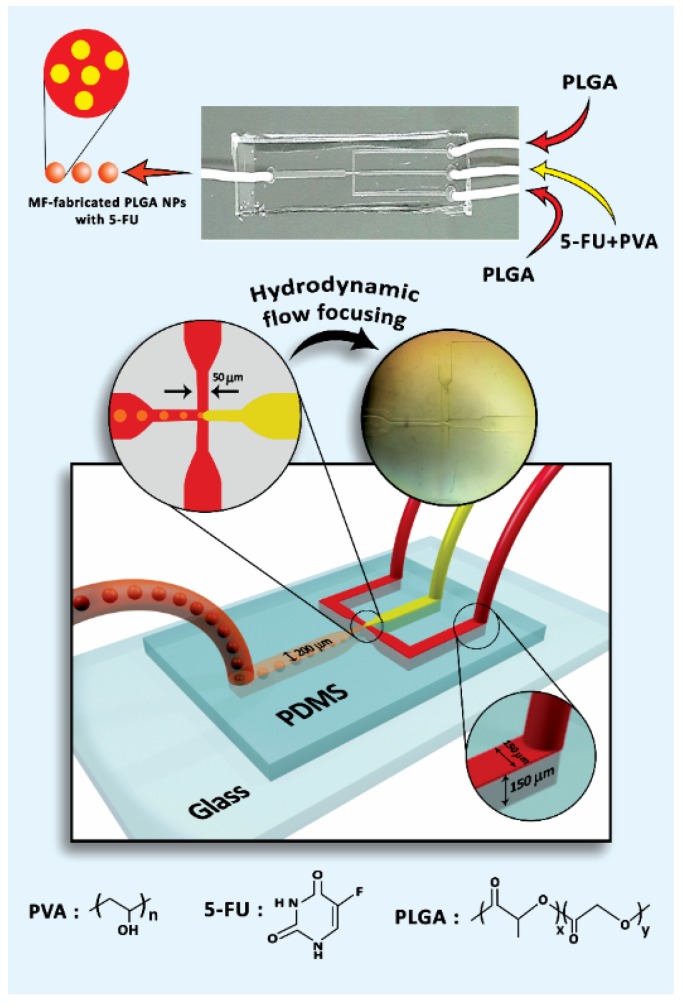
Schematic of microfluidic (MF) device setup.

A silicone-based chip was prepared according to the literature [28]. In brief, the procedure to produce the MF chip with a fork-shaped pattern was as follows: (1) designing the pattern by AutoCAD 2016 and printing it on a high contrast film as photomask, (2) providing a thin layer of SU-8 as photoresist polymer on a silicon wafer by a spin-coating process and soft-baking of the coated SU-8, (3) adhering the photomask to the baked SU-8-coated silicon wafer, (4) exposure to ultraviolet (UV) rays and post-exposure bake, (5) using an SU-8 developer called 1-methoxy-2-propyl acetate and washing by isopropyl alcohol for non-crosslinked portions removal, which formed the master mold, and (6) preparing the silicone-based chip by curing with polydimethylsiloxane (PDMS) on the master mold [29]. Subsequently, the chip was treated with oxygen-plasma [30] to stick it on the glass and prevent rapid swelling under exposure of solvents like acetone [31]. Finally, in order to fabricate the polymer nanocarriers, polytetrafluoroethylene (PTFE) tubes (inner diameter of 1 mm) were inserted into the MF device. The overall schematic of the microfluidic chip alongside three inlets and one outlet is shown in Figure 1.

### 2.3. Preparation of MF-Fabricated PLGA NPs Containing 5-FU

To fabricate the polymeric NPs, different amounts of PLGA (1, 2, 3 and 5 mg) in 10 mL of dichloromethane/acetone [80/20 (*v*/*v*)] at room temperature, 100 µg of 5-FU powder based on IC50 assay in 10 mL of DMSO at ambient temperature and 1.5 mg of PVA in 10 mL of distilled water at 80 °C were dissolved. In general, IC50 defined as a half-maximal inhibitory concentration of drug which is needed to stop a given biological process. Hereby, this value was measured for 5-FU effectiveness on Caco2 cells at the different drug concentrations to load into the PLGA NPs. Consequently, the lower concentration was considered for further evaluation owing to minimum cytotoxicity for intact cells. A similar trend was also seen for IC50 values of SW-480 with the same drug concentrations.

The mixture of 5-FU and PVA (as surfactant) solutions [1% (*v*/*v*)] was inserted in the middle microchannel as a dispersed phase and PLGA solution as a continuous phase was injected into the side microchannels (Figure 1). According to the hydrodynamic flow focusing phenomenon [32], the oil and water phases contact each other, leading to droplet generation via the emulsification mechanism [33]. In this line, the flow rate of polymer solution (Q**_polymer_**) was set at 5 mL/h and this variable for the drug-surfactant solution (Q**_drug_**) was selected in the range of 0.5 to 2 mL/h to investigate the effect of various flow-rate ratios (Q**_polymer_**/Q**_drug_**) on particle size. It is worth noting that the silicone-based chip was gradually swollen while producing the PLGA NPs and it should be replaced with new ones to be reproducible for the samples. To remove the acetone and DCM completely from the samples, they were washed at least 3 times with distilled water and allowed to dry in ambient temperature for 48 h [34].

### 2.4. Dynamic Light Scattering Studies

The particle-size distribution and colloidal dispersion stability of the samples prepared by the MF technique were characterized utilizing a dynamic light scattering [DLS, model Plus PALS 90, Brookhaven Co., Holtsville, USA] associated with zeta potential analyzer. In this line, the solution from the outlet of MF chip was sonicated in a water bath for 30 min and then transferred to a cuvette for the measurements at room temperature.

### 2.5. Field-Emission Scanning Electron Microscopy Studies

The morphology of the samples was observed using a field-emission scanning electron microscopy (FE-SEM model S-4160, Hitachi Co., Tokyo, Japan] with 6000× and 30000× magnifications. In this line, the outlet solution from the MF chip was centrifuged at 4000 rpm at room temperature for 20 min. Afterward, the supernatant was poured on a flat surface and finally sputter-coated with a thin layer of gold for imaging with high resolution.

### 2.6. 5-FU Release from MF-Fabricated PLGA NPs Alongside Kinetic Models Analysis

The encapsulation efficiency (EE) was defined as the percentage of encapsulated 5-FU with respect to the total amount of the drug in PLGA NPs prepared via freeze-drying [model Alpha 1-2 Lo Plus, Christ Co., Osterode, Germany]. To measure the drug release amounts, the MF outlet solutions were first centrifuged at 14,000 rpm, 4 °C for 20 min. Then the supernatants were taken to measure the drug concentration using ultraviolet-visible spectrophotometry (UV-vis, model NanoDrop^®^ND-1000, Thermo Fisher Scientific Inc., Victoria, Australia). Using 5-FU calibration curve, which was the drug absorbance at a maximum absorption wavelength of 265 nm as a function of concentration, the total amount of the drug, its encapsulation and cumulative release were calculated based on Equations (1)–(3).

(1) $$Drug\;content\;\left(\%\right)=\frac{Amount\;of\;drug\;in\;PLGA\;NPs}{Amount\;of\;PLGA\;NPs}\times 100$$

(2) $$\begin{array}{c} Encapsulation\;efficiency\;\left(\%\right)\\ =\frac{Total\;drug\;loaded\;into\;PLGA\;NPs-Free\;nonentrapped\;drug}{Total\;drug}\times 100 \end{array}$$

(3) $$\begin{array}{cc} Drug & release=\frac{\begin{array}{ccccc} Amount & of & drug & release & \left(mg\right)\times 100 \end{array}}{\begin{array}{ccccc} Total & amount & of & drug & \left(mg\right) \end{array}} \end{array}$$

Three mathematical models including zero-order, first-order and Higuchi were investigated in this work to study the release kinetics of neat 5-FU and MF-fabricated PLGA NPs (0.2)/5-FU.

### 2.7. Cell Culture

Colon cell lines, Caco2 and SW-480, including human colorectal adenocarcinoma, were cultured in Roswell Park Memorial Institute medium (RPMI) [35] and (DMEM) [36] respectively supplemented with 10% FBS. Hereby, the cell lines were cultured and then treated with the samples in an incubator at 37 °C with 5% CO_2_.

### 2.8. MTT Assay

The cytotoxicity of free 5-FU (0.01 µg/mL), MF-fabricated NPs based on PLGA with concentrations of 0.1% and 0.2% (*w*/*v*) containing the drug and control (the cells feeding with culture medium), was determined by MTT assay. To assess the relative cell viability of the samples, the cell lines were seeded on into a 96-well culture plate with a density of 5×10^3^ cells/well. After 24 h, 48 h and 72 h of incubation, the amounts of formazan representing the alive cells were measured using an ELISA reader (EL-312, BioTek Instruments Inc., Winooski, VT, USA) at 540 nm absorbance. Afterward, the relative cell viability was obtained by dividing the optical density (OD) of each sample (recording changes in absorbance at 540 nm and a reference wavelength of 620 nm) by OD of control based on Equation (3) [37]:

(4) $$Relative\;cell\;viability\;\left(\%\right)=\frac{OD_{sample}}{OD_{control}}\times 100$$

### 2.9. Flow Cytometry

Regarding the cell death, a flow cytometer device [model BD FACSCalibur^TM^ (BD Biosciences, San Jose, CA, USA)] was utilized to consolidate the information related to 1000 μL cells/min [38]. By sorting the high-performance heterogenic mixture of the cell population with 100 µL in volume, individual cells were separated from the mixture. Based on the difference between the size and behavior of the inner structure of the cells against 5-FU, the laser light at a wavelength of 448 nm was scattered. Accordingly, two main scattered lights, such as forward scattered (FSC) and side scattered (SSD), were obtained for providing the scattered blots containing amounts of cells and their fluorescent level information. In this test, pyridinium iodide (PI, Sigma-Aldrich, Darmstadt; Germany) was used to stain the DNA of cells for determining the dead and living cells. Hereby, four groups including the MF-fabricated NPs containing 0.1% and 0.2% (**w*/*v**) of PLGA, neat 5-FU and control (cells in their culture medium) were considered for both cell lines. The following procedure was employed to prepare the samples for flow cytometry: First, 1 mL of cell suspension containing 5 × 10^5^ cells/mL was poured into 6-well plates and 2 mL of culture medium was added to the wells. Subsequently, the cells were incubated at 37 °C for 48 h. After that, the wells were washed with 6 mL of PBS, followed by centrifugation at 2000 rpm for 10 min and after the addition of 10 µL of PI to the wells, incubated on ice cubes for 30 min. Then PBS (1 mL) was added to the samples followed by carrying out a flow cytometry test three times; subsequently, the obtained data were analyzed by FlowJo software (v10.6.1). Gating [39], a strategy for categorizing the cells with the same characteristics used in flow cytometry results interpretation.

### 2.10. Immunofluorescence Assay

The cell death was determined morphologically via 4′, 6-diamidino-2-phenylin-dole (DAPI), which is a DNA-specific probe for the formation of a fluorescent complex by attaching in the minor grove of A-T rich sequences of DNA. Hereby, Caco2 and SW-480 cell lines were seeded in 6-well plates including a coverslip with 1 × 10^5^ cells/well and cultured at 37 °C for 24 h. The cell lines in the exposure of cultivation mediums were considered as the control groups. They were incubated in the presence of PLGA NPs for 4 h and were taken into account for the comparison. Then the samples were fixed with 4% paraformaldehyde in PBS at room temperature (25 °C) for 15 min, stained with 0.2 µg/mL DAPI (Sigma-Aldrich, St. Louis, MO, USA) in PBS at 25 °C for 15 min and washed two times with PBS and water. The prepared samples were placed on the lams and the DAPI-stained cells were examined using a fluorescent microscope.

### 2.11. Statistical Analysis

The results were expressed as mean ± SD (standard deviation). The two-way analysis of variance (ANOVA) was used to evaluate the significance of the results with p-value in which those with values less than 0.05 and more than 0.1 have importance and unimportance, respectively, for the comparison.

## 3. Results and Discussion

### 3.1. The Role of Flow Rates of Polymer and Drug Solutions on PLGA/5-FU Droplets Generation

**Figure 2:**
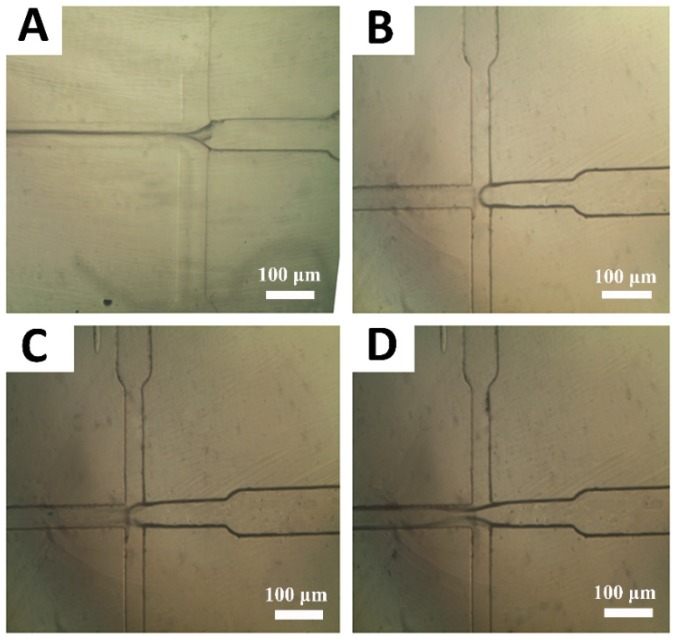
Effect of flow rates ratio (Q_polymer_/Q_drug_) on the production of poly (lactic-co-glycolic) acid (PLGA) droplets: (**A**) 10, (**B**) 5, (**C**) 3.3 and (**D**) 2.5. To achieve these values, the drug flows in the middle channel were adjusted at 0.5 mL/h, 1 mL/h, 1.5 mL/h and 2 mL/h. Also, the polymer flow in the side channels was fixed at 5 mL/h.

To minimize the size of emulsion droplets, different ratios of the polymer to drug/surfactant flow rates were investigated. Figure 2A–D depicts the inverted optical microscope images from the hydrodynamic flow focusing zones for different Q_polymer_/Q_drug_ values as shown in 10 [Figure 2A], 5 [Figure 2B], 3.3 [Figure 2C] and 2.5 [Figure 2D]. The size and size distribution of the droplets could be easily controlled by changing the flow rates of the two phases. By considering Figure 2A–D, it has been found that the flow-focusing phenomenon occurred while adjusting the drug flow (middle channel) and the polymer flow (side channels) with amounts of 0.5 mL/h and 5 mL/h, respectively. In other words, by controlling these values, the flow rates ratio of polymer to the drug was equal to 10.

### 3.2. Morphology, Size and Size Distribution of NPs

**Figure 3:**
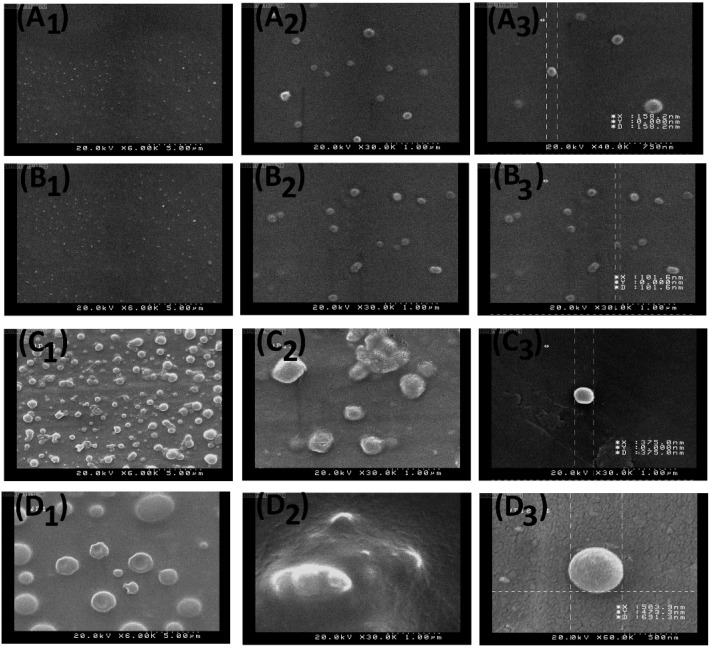
FE-SEM images of the nanoparticles (NPs) with different concentration ratios: (**A_1_**) 0.1% of PLGA at 6000×, (**A_2_**) 0.1% of PLGA at 30,000×, (**A_3_**) a size representation of 0.1% of PLGA NP at 40,000×, (**B_1_**) 0.2% of PLGA at 6000×, (**B_2_**) 0.2% of PLGA at 30,000×, (**B_3_**) a size representation of 0.2% of PLGA NP at 30,000×, (**C_1_**) 0.3% of PLGA at 6000×, (**C_2_**) 0.3% of PLGA at 30,000 X, (**C_3_**) a size representation of 0.3% of PLGA NP at 30,000×, (**D_1_**) 0.5% of PLGA at 6000×, (**D_2_**) 0.5% of PLGA at 30,000× and (**D_3_**) a size representation of 0.5% of PLGA NP at 60,000×.

Figure 3A_1_–D_3_ shows the FE-SEM micrograph images of the samples including MF-fabricated NPs [0.1% (*w*/*v*)] [Figure 3A_1_–A_3_], MF-fabricated NPs [0.2% (*w*/*v*)] [Figure 3B_1_–B_3_], MF-fabricated NPs [0.3% (*w*/*v*)] [Figure 3C_1_–C_3_] and MF-fabricated NPs [0.5% (*w*/*v*)] [Figure 3D_1_–D_3_]. Evidently, by increasing the PLGA solution concentration from 0.1% to 0.5% (*w*/*v*), the size of NPs was increased.

**Figure 4:**
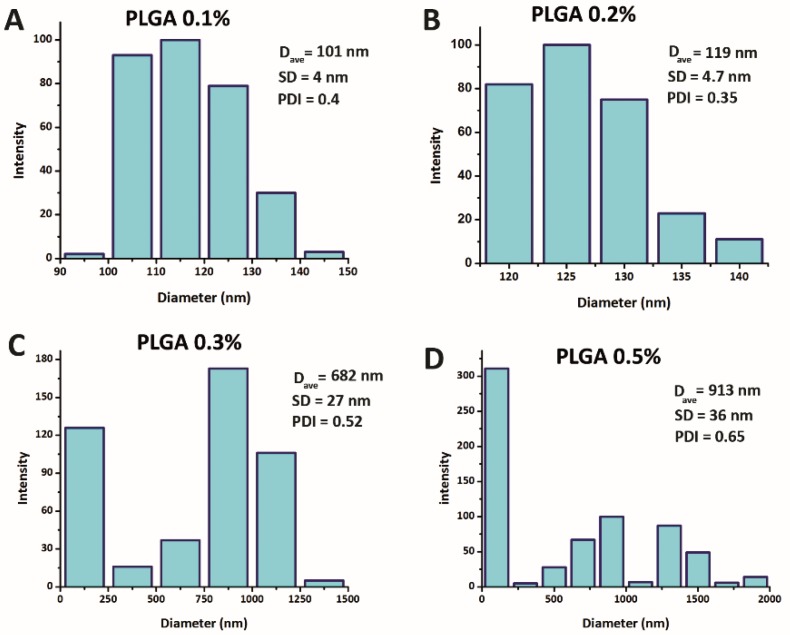
NPs size and their size distribution curves based on dynamic light scattering (DLS) at different PLGA concentrations: (**A**) 0.1 (% *w*/*v*), (**B**) 0.2 (% *w*/*v*), (**C**) 0.3 (% *w*/*v*) and (**D**) 0.5 (% *w*/*v*). (The p-value for all data was less than 0.05).

Figure 4A–D illustrates the size and distribution of PLGA/5-FU NPs based on DLS results. Accordingly, four different concentrations of PLGA, i.e., 0.1 (% *w*/*v*), 0.2 (% *w*/*v*), 0.3 (% *w*/*v*) and 0.5 (% *w*/*v*), were selected. Obviously, by increasing the PLGA concentration, the sizes of NPs were increased and their size distribution curves have undergone an irregularity alongside a broad shape. Upon morphology, size and size distribution of NPs, results showed that the NPs were enlarged by increasing the PLGA solution concentration from 0.1% to 0.5% (*w*/*v*). This observation could be related to the more vulnerability of PLGA/5-FU NPs to form aggregates through hydrodynamic flow focusing zone in MF chip. Based on the concentrations of polymer with the amounts of 0.1% and 0.2% (*w*/*v*) containing the drug, the specific method led to fabricate the NPs through the MF chip with mean sizes of 101 nm and 119 nm, respectively alongside the PDI value less than 0.5. Furthermore, a different trend was observed in Figure 4D owing to the rearrangement of the polymer chains and agglomeration depending on the structure of the flow field.

### 3.3. 5-FU Loading and Its Release Kinetics Studies

To plot the 5-FU calibration curve, different concentrations of 5-FU were dissolved in DMSO and the obtained relationship between the absorbance (A) and concentration (C) of the drug solution according to the Beer-Lambert law was represented in Equation (4) as follows:

(5) A=0.003×C+0.1426

**Figure 5:**
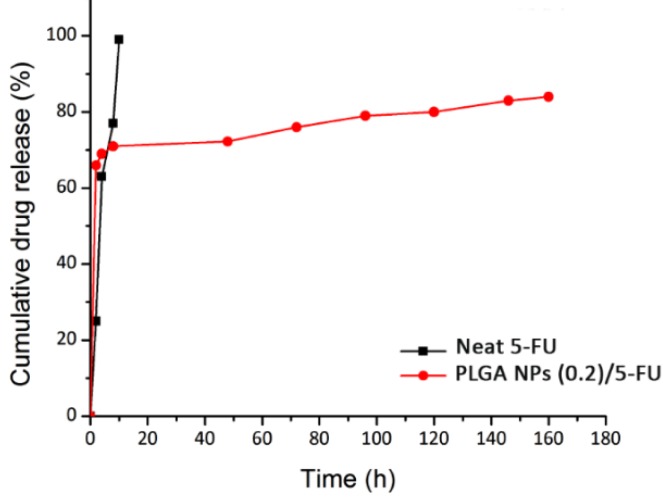
In vitro release profiles of neat 5-FU and PLGA NPs (0.2)/5-FU during 165 h.

The release profiles of neat 5-FU (squared line) and 5-FU loaded PLGA NPs (0.2) (circled line) are represented within PBS (pH = 7.4, 37 °C) in Figure 5. As is seen, a rapid initial release for both samples happened. Regarding the drug-loaded NPs, occurring this trend was due to the formation of weak bonds between drug and polymer and/or the superficial adsorption of drug molecules onto the surface of NPs. Moreover, by passing through the time, the drug release profile for polymer nanocarriers was undergone a plateau status which was due to the diffusion of the drug molecules dispersed into the polymer matrix. The reason for drug encapsulation into the PLGA NPs was to control and prolong its release due to the short half-life (t_1/2_) of 5-FU ranging from 10 to 20 min.

Prior to use Equation (3) for calculating the cumulative release of 5-FU from the sample, the total drug content was obtained by Equation (1) which was equal to 0.5 mg. On the other hand, to show the MF performance in view of EE%, Equation (2) was employed and the achieved data were repeated three times (Nv = 3). The EE with the amount of 95% ± 3.95% was calculated for the MF system used and the result was compared to the conventional method (Table 1). The overall performance of MF technique in this work showed remarkable properties especially EE term. Table 2 depicts the regression coefficients (r^2^) of three different kinetic models corresponding to the experimental data of 5-FU release. For the study of 5-FU loading and analysis of the drug release kinetic, Equation (5) has good correspondence to the experimental data with a regression coefficient (r^2^) of 0.95 at the maximum absorbance wavelength of 295 nm. Regarding Figure 5, the red line (release profile of 5-FU) fitted with first-order profile illustrates a more controlled release of anti-cancer drug for 165 h plus high EE of 98.95%. In addition, in the case of neat 5-FU as in Table 2, the obtained value was 0.95 for the zero-order model, resulting in the best fit compared to first-order and Higuchi models. It could be related to dissolving the free molecules of the drug in the release medium and the direct relation of this phenomenon with time. Conversely, by considering the r^2^ value of MF-fabricated PLGA NPs (0.2)/5-FU, its amount was 0.96 which followed the Higuchi model. This could be due to the formation of PLGA layers around the 5-FU molecules, which led to the release of drug attributing to the square root of time.

### 3.4. Investigating Cytotoxicity of by MTT

**Figure 6:**
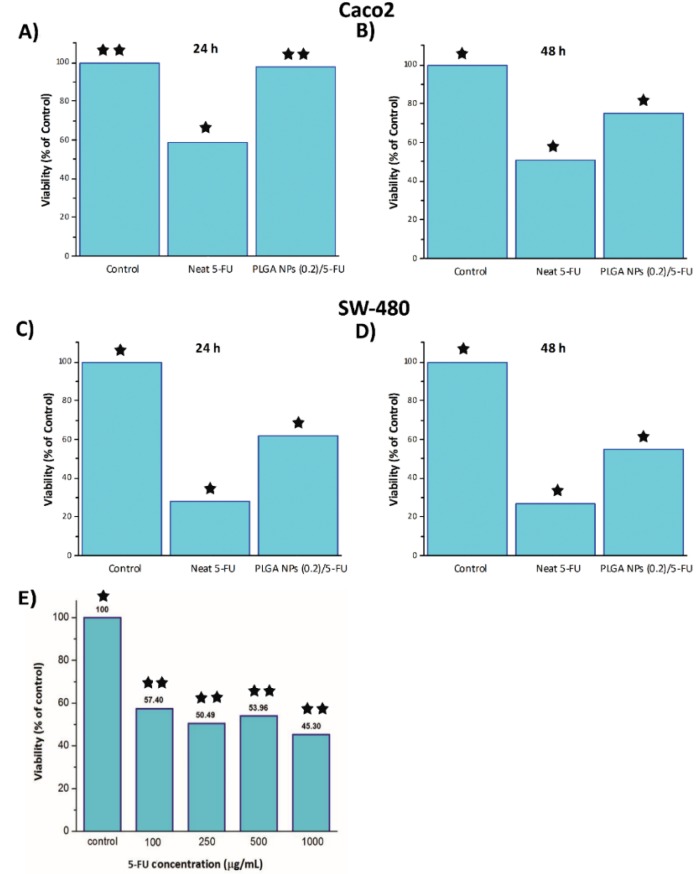
MTT cytotoxicity assay results: Caco2 (**A**) 24 h, (**B**) 48 h and SW-480 (**C**) 24 h and (**D**) 48 h. Also, IC50 of 5-FU dosage in various concentrations through cell culture (**E**). [One star stands for p-value less than 0.05 and two stars stand for p-value more than 0.1].

Figure 6A–D demonstrates the MTT results of neat 5-FU, PLGA NPs (0.2)/5-FU and control samples containing Caco2 [Figure 6A,B] and SW-480 [Figure 6C,D] cell lines in their cultivation media. Obviously, the viability of these cancer cell lines in the exposure of neat 5-FU and MF-fabricated PLGA NPs (0.2)/5-FU was decreased by increasing the time. Also, as mentioned earlier, the IC50 of drug at different concentrations was studied and shown in Figure 6E. As is seen, the difference between IC50 values of the drug concentrations with 250 and 500 µg/mL was negligible. Besides IC50, the half maximal effective concentration (EC50) of the drug can be considered as another main parameters and is commonly used.

### 3.5. Flow Cytometry and DAPI Staining

**Figure 7:**
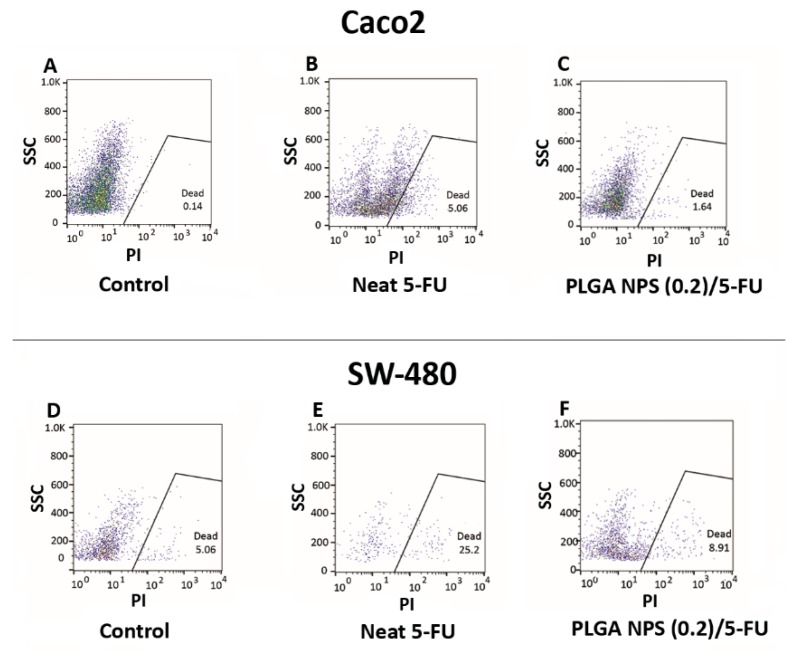
Flow cytometry results for Caco2 cell line: (**A**) control, (**B**) neat 5-FU and (**C**) PLGA NPs (0.2)/5-FU, besides for SW-480 cell line: (**D**) control, (**E**) neat 5-FU and (**F**) PLGA NPs (0.2)/5-FU.

The cell death results of Caco2 and SW-480 colon cancer cell lines were evaluated on three groups including neat 5-FU, MF-fabricated PLGA NPs (0.2)/5-FU and the cells in their culture medium as control samples in order to confirm the MTT outcomes. Figure 7A–F illustrates the flow data of the samples after 48 h. Based on Figure 7A–C, the percentages of cell death for Caco2 were 0.14%, 5.06% and 1.64% for control, neat 5-FU and MF-fabricated PLGA NPs (0.2)/5-FU respectively. Additionally, these values for the SW-480 cell line regarding the samples with the same order mentioned above were 5.06%, 25.20% and 8.91% [Figure 7D–F].

**Figure 8:**
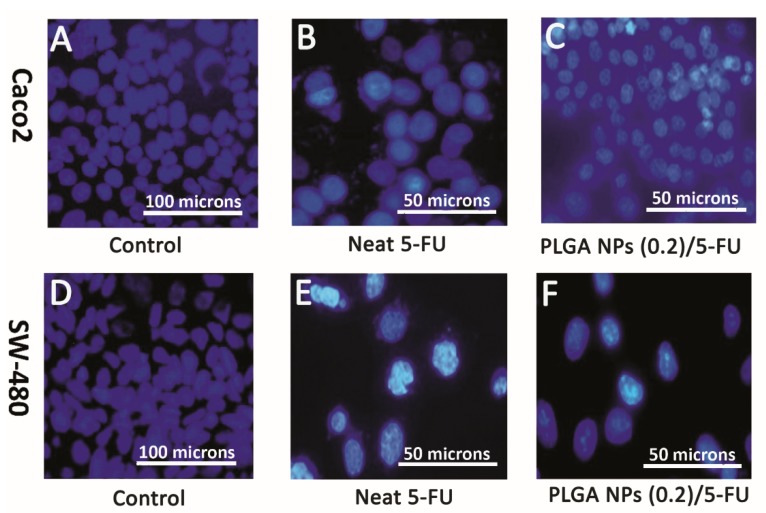
Representative fluorescence micrographs (DAPI, blue) showing morphologically death of Caco2 and SW-480 cell lines after DAPI staining: (**A**,**D**) control [magnification 200×], (**B**,**E**) neat 5-FU and (**C**,**F**) PLGA NPs (0.2)/5-FU [magnification 400×].

For further evaluation, DAPI staining was carried out on these two cell lines in the presence of control, neat 5-FU and MF-fabricated PLGA NPs (0.2)/5-FU samples. Figure 8A–F shows the immunofluorescence microscopic images, explaining the morphological death of Caco2 and SW-480 cell lines. Obviously, the amounts of the lightened cells were decreased in the presence of neat 5-FU and PLGA NPs/5-FU. By comparing the flow data of Figure 7B,C, the level of cell death for the neat 5-FU was more significant than that of PLGA NPs/5-FU attributing to the polymeric cover around the drug molecules which led to the controlled release of the drug [43]. The same trend could occur for the SW-480 with 6.6-fold more than that the cell death for Caco2. This behavior could be referred to as the non-metastatic property or less resistance of SW-480 cell line against 5-FU treatment compared to Caco2 [44,45].

## 4. Conclusions

In this study, PLGA NPs [0.2 (% *w*/*v*)] containing 5-FU (100 µg/mL) were successfully prepared using the MF system associated with a fork-shaped pattern embedded on a silicon chip. The best flow-rate ratio of the polymer to the drug-surfactant solutions was equal to 10, thereby obtaining the relatively uniform NPs with narrow PDI. Subsequently, drug release studies showed that drug encapsulation led to more controllable release than a neat 5-FU. Also, flow cytometry and DAPI staining confirmed that the MF-assisted PLGA NPs (0.2)/5-FU were able to kill cancerous colon cells at a gradual rate and safe drug dosage. In conclusion, the obtained results are promising for using this system in colon cancer treatment applications.

## Figures and Tables

**Table 1:** A comparison between MF-based PLGA NPs and the NPs produced by other conventional methods in terms of size, polydispersity index (PDI) and encapsulation efficiency (EE) values reported in the literature.

Formulation/Method	NPs Size (nm)	EE (%)	PDI	Reference
5-FU loaded poly (3-hydroxybutyrate-co-3-hydroxyvalerate) (PHBV)/PLGA NPs/emulsion	~135	~43	≤1	[40]
5-FU Loaded PLGA and PCL NPs a simple/emulsion method [without poly (ethylene glycol) (PEG)]	~24	~32	0.137	[41]
5-FU-PLGA NPs/nanoprecipitation	~133	~40	0.352	[42]
5-FU loaded PLGA NPs/MF technique	~101	~95	0.083	Current study

**Table 2:** Regression coefficients of different mathematical models fitted to the release of 5-FU in the neat form and from MF-fabricated PLGA NPs [0.2 (% *w*/*v*)].

Sample	Zero-Order	First-Order	Higuchi
Neat 5-FU	0.95	0.64	0.65
MF-fabricated PLGA NPs (0.2)/5-FU	0.63	0.87	0.96
